# Prosecutorial decision-making regarding offenders’ social reintegration programs in intimate partner violence cases. A Portuguese study

**DOI:** 10.1371/journal.pone.0269820

**Published:** 2022-06-16

**Authors:** Paulo Vieira-Pinto, José Ignacio Muñoz-Barús, Tiago Taveira-Gomes, Maria João Vidal-Alves, Teresa Magalhães

**Affiliations:** 1 Institute of Forensic Sciences—Department of Forensic Sciences, Pathology, Gynecology and Obstetrics, Pediatrics, University of Santiago de Compostela, Santiago de Compostela, Spain; 2 TOXRUN–Toxicology Research Unit, University Institute of Health Sciences, CESPU, CRL, Gandra, Portugal; 3 Faculty of Medicine, University of Porto, Porto, Portugal; 4 CINTESIS—Center of Health Technology and Service Research, University of Porto, Porto, Portugal; 5 Fernando Pessoa University, Porto, Portugal; Sapienza, University of Rome, ITALY

## Abstract

Intimate partner violence is one of the most challenging and demanding problems that the criminal justice system has to face. Given the severe consequences of intimate partner violence, it is imperative that intervention from the criminal justice system, regarding perpetrators, be effective to prevent further victimization and recurrences. In Portugal, it is up to the state prosecutor to decide which cases will be subject to a social reintegration program as a pretrial diversion program. This study aims to explore the variables that might influence the state prosecutor’s decision-making process. We have examined 283 intimate partner violence cases in which provisional suspension of criminal proceedings was applied. The decision as to whether defendants should be referred for social reintegration program attendance (G1) or not (G2) was made by the state prosecutor. Differences between G1 and G2 were identified: the victim’s age, couple living in a current relationship, drug-addicted defendant, intimate partner violence child exposure. However, defendants’ unemployment and drug abuse were the only two variables identified as a determinant for state prosecutor decisions. We believe that the effectiveness of state prosecution decision-making would benefit from: (a) systematically taking into account all intimate partner violence risk factors; (b) an index or checklist detailing what science reveals useful in intimate partner violence offenders’ social reintegration; (c) rehabilitation solutions based on the needs of each offender instead of a “one-size-fits-all” approach.

## Introduction

The severe consequences of intimate partner violence (IPV) for victims, families, and the community as a whole, are widely described in the literature [1, 2]. Particularly relevant, to deter this kind of violence, and avoid or reduce its serious outcomes, is the early detection and intervention, not only in legal terms but also from the therapeutic/rehabilitation and social reintegration perspectives.

If IPV is considered a pathology of the interaction between couples, the intervention needs to include the victim, as well as the alleged offender. However, the focus of the intervention, namely for therapy/rehabilitation, has mainly been the victim. This is fundamental but not enough to prevent revictimization (when a victim is repeatedly exposed to violent behavior by an intimate partner [3]). The intervention with offenders is fundamental despite being infrequent in most countries [4, 5]. This may be due to multiple factors, including not only the judicial decision-making process but also the defendant’s availability and motivation for the procedures (which is the cause of a significant number of drop-outs) [6, 7]. It was found that the severity of the injuries is associated with the offender’s tendency to repeat aggressions [8]. Therefore, the therapy/rehabilitation and social reintegration processes are particularly important, especially in these more severe cases.

Several studies suggest that prosecution and actual conviction in IPV cases rarely occur [9–12]. Only 14.9% of defendants in Portugal were prosecuted in 2020, with 2.4%of those convicted [45]–and 17.2% of those sentenced to jail [46]. Furthermore, convictions seem to be inefficient and ineffective towards reoffending and recidivism [8, 13, 14], besides the personal, social, and economic costs associated with the imprisonment system [13, 15].

Thus, crime prevention, namely tertiary prevention [16–18], which includes Social Rehabilitation Programs (SRP), is imperative for offenders. It focuses on preventing revictimization and recidivism and avoids incarceration [16]. Strategies may include treatments (e.g., cognitive-behavioural therapy [19, 20], or substance abuse addiction therapy [21]), community-based programs [16, 22] or other suitable services [23, 24]. The interventions should always be guided by the principles of risk, need, and responsivity [25].

### The Portuguese case

**Fig 1:**
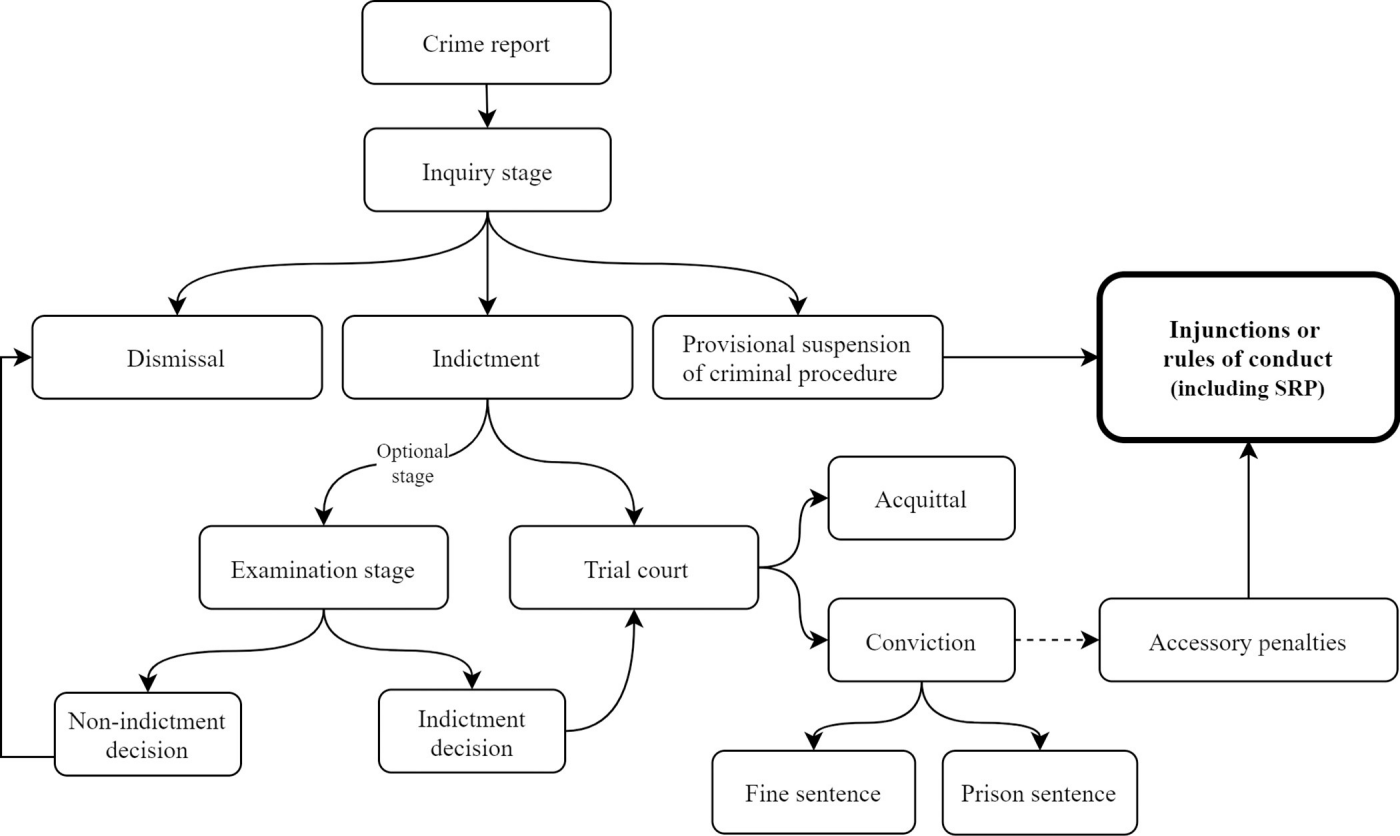
Phases of criminal procedures.

In Portugal, IPV is considered a domestic violence crime of public nature (article 152.° of the Portuguese Criminal Code). This means that from the moment the public prosecutor’s office suspects a possible case, an inquiry is opened (article 263.° of the Criminal Procedure Code). The investigation is carried out by the state prosecutor who leads the inquiry. After gathering evidence, they will decide on the case filing, whether to proceed with the indictment or the provisional suspension of criminal proceedings (PSCP) with the application of alternative measures. The state prosecutor will proceed with the indictment if the defendant does not consent to the PSCP’s implementation (Fig 1).

The PSCP is used as an integrated consensual solution between the involved parties, targeting the defendant’s resocialization and the prevention of revictimization [26]. After PSCP implementation, the defendant must accept a set of injunctions or rules of conduct for a maximum period of 5 years. Twelve injunctions exist, according to article 281.° of the Criminal Procedure Code, namely the attendance of certain programs or activities, where the SRP are included. If the defendant complies, the case is dismissed pending the investigative judge’s agreement. Between 2013 and 2018, PSCP increased in Portugal by 60% [27]. However, its implementation in IPV cases is low (17%) [26].

The official Portuguese SRP is named *Program for Domestic Violence Offenders* (PAVD), designed to address only male defendants/convicted of IPV with a current or past relationship with a female victim [28]. The PAVD is executed by the *Directorate-General for Reintegration and Prison Services* (DGRPS), whose mission is to develop policies on criminal prevention, supervising the compliance of criminal measures decreed by the CJS, targeting the defendant´s social reintegration. PAVD aims to promote the perpetrators’ awareness and assumption of responsibility for their violent behaviour, as well as the use of alternative strategies to reduce reoffences and recidivism [28, 29]. This program uses a psychoeducational group intervention delivered in 20 sessions, in which several structural relational issues associated with IPV are addressed. It has a minimum duration of 18 months and requires the defendant to abide by the imposed rules and measures, which must be verified. It can be court-mandated as an accessory penalty or as a result of the PSCP agreement. It is also used, mandatorily, as an accessory penalty to offenders convicted to a prison sentence. PAVD has been tested and presents positive results [30]. DGRSP may also decide to send offenders for SRP implemented by non-governmental organizations [31–33]. Offenders are free to apply and attend these programs voluntarily.

In Portugal, not all PSCP cases get referred to SRP. The referral is decided by the state prosecutor on a case-by-case basis according to their own criteria [34]. However, the literature indicates that to decide whether a specific IPV defendant is to be recommended for this type of program, a holistic assessment of both offender and victim should be performed, taking into account [35–38]: age, education level, employment status, interpersonal relationship, type of inflicted violence, and substance abuse.

The literature in Portugal on the state prosecutor decision-making process regarding SRP implementation is almost nil [34]. Thus, drawing on the theoretical background outlined above, the current study aims to identify variables that may influence state prosecutor decision-making regarding the submission of defendants for SRP by the DGRSP.

## Materials and methods

**Fig 2:**
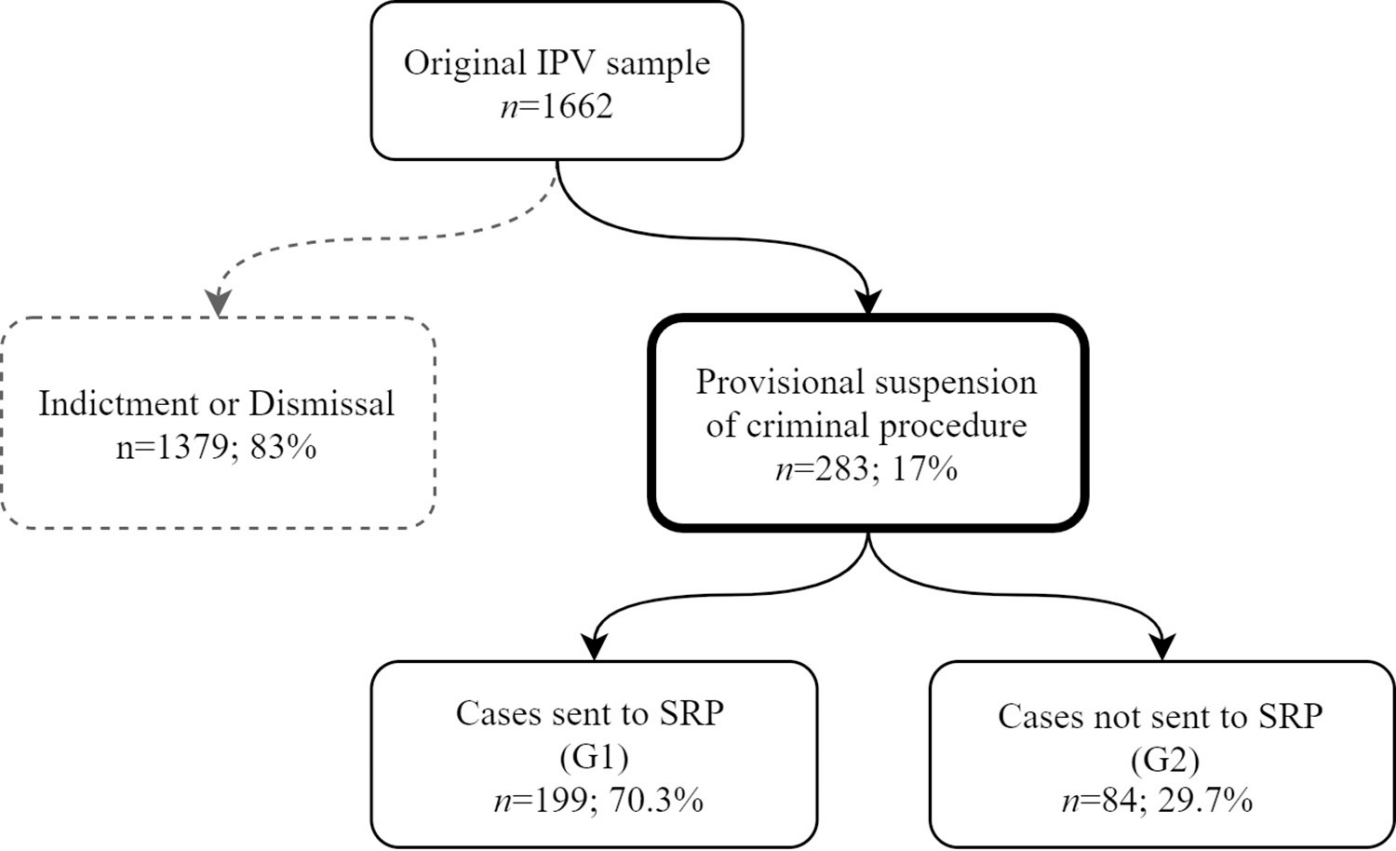
Cases’ selection.

Data were obtained from Porto County District Prosecutor’s Office database, selected according to a non-randomized convenience sampling process, between 1st May 2019 and 1st January 2020 (Fig 2), following previous studies on these cases [8, 26].

Data were obtained through IPV police reports and filled in by information provided by the parties involved. The inclusion criteria of these previous studies were: (a) IPV case (between former or current intimate partners, dating, married or analogous, regardless of cohabitation); (b) victim: female aged 16 or older; (c) defendant: male, aged 16 (minimum age for criminal liability) or older; (d) cases in which PSCP was applied.

In the current study, a retrospective cohort design was used, focusing on those where PSCP was implemented. The data collection further encompassed a crosscheck of the DGRSP database, with a list of male defendants who accepted the PSCP application as well as the injunctions and rules of conduct decreed by judicial authorities (e.g. order the defendat to avoid all personal contact with the victim; Order the defendat to move out of and/or stay away from the victim´s home, business, school or other locations; order the defendat to stop all harassing, threatening and violent behavior; order the defendat to attend specific programs or activities). In case the defendant abides by such rules, proceedings shall be dismissed; if not, they shall continue to Trial.

IPV cases were selected concerning the period between January 1, 2010, and December 31, 2013 (*n* = 1.662). These cases were subsequently cross analyzed with the Public Prosecution Service’s IPV database, to check whether the selected cases had undergone PSCP. Two groups were then created, considering this last feature: G1 –With PSCP (*n* = 283); G2 –Without PSCP (*n* = 1.379). From the 1662 IPV cases, 283 were chosen in which the defendant agreed to the PSCP and, as a necessary consequence, the imposition of injunctions by the state prosecutor. This sample was then divided into two groups: G1, including cases sent to SRP by DGRPS (*n* = 199); G2, including cases with other types of injunctions (*n* = 84)–Fig 2.

A comparative study was then performed to identify differences between both groups (victims and defendants’ characterization, and violence-related variables), looking for a pattern of variables that may impact the state prosecutor’s decision-making process.

Statistical analysis was conducted using the R programming language R—version 4.0.5 [39]. In addition to the descriptive analysis, hypothesis testing for the association of characteristics with re-entry was carried out using Chi-Square. The pattern of missing data was assessed using Little’s Missing Completely at Random (MCAR) test using the LittleMCAR package [40]. The analysis was performed on imputed data using the Multivariate Imputation by Chained Equations (MICE) package [41]. Categorical variables were imputed using a proportional odds model and continuous variables using unconditional mean imputation. All variables presented were considered in the imputation model. Data imputation was repeated 100 times. To assess the reliability of the results, the same analysis was performed with the removal of incomplete cases relevant for each test. Significance was calculated to be *p*<0.05.

Access and permission to use the prosecutor administrative records database was granted by Porto County District Prosecutor’s Office. The study protocol was approved by the Ethics Committee for Health of *Centro Hospitalar de São João / Faculty of Medicine*, *University of Porto*, Porto. All guarantees of anonymity given were honored.

## Results

The victims and defendants’ characterization are described in Table 1, and the relationship between the couple, as well as children´s exposure to violence, are described in Table 2. Victims average age was: (a) G1–44.06 years old (Min = 18, Max = 86; *SD* = 12.42); G2–41.37 years old (Min = 20, Max = 74; *SD* = 10.97). Defendant´s average age was: G1–47.12 years old (Min = 19, Max = 86; *SD* = 12.38); G2–44.29 years old (Min = 21, Max = 75; *SD* = 12.23). Significant statistical association was found regarding: victim’s age (*χ*^2^ = 18.81; *p* = 0.043); couple living in a current relationship (*χ*^2^ = 6.19; *p* = 0.045); drug addicted defendant (*χ*^2^ = 17.47; *p* = 0.002); IPV children exposure (*χ*^2^ = 9.75; *p* = 0.045).

**Table 1:** Victims and defendants demographics and risk factors.

		Victims				p*	Defendants				p*
		G1 (n = 199)		G2 (n = 84)			G1 (n = 199)		G2 (n = 84)		
		*n*	*%*	*n*	*%*		*n*	*%*	*n*	*%*	
Age (years)	≥ 16–30	32	16.1	13	15.5	0.043 (0.043)	167	1.0	15	17.9	0.128 (0.128)
	31–40	39	19.6	31	36.9		372	16.6	18	21.4	
	41–50	74	37.2	21	25.0		520	30.2	22	26.2	
	51–60	34	17.1	16	19.0		356	24.6	20	23.8	
	≥ 60	18	9.0	3	3.6		184	6.5	8	9.5	
	Missing	2	1.0	0	0.0		11	5.5	1	1.2	
Employment status	Employed	66	33.2	29	34.5	1 (1)	71	35.7	33	39.3	0.246 (0.246)
	Unemployed	69	34.7	30	35.7		78	39.2	24	28.6	
	Missing	64	32.2	25	29.8		50	25.1	27	32.1	
Alcohol abuse	Yes	*n*.*a*					102	51.3	43	51.2	0.958 (0.958)
	No						65	32.7	29	34.5	
	Missing						32	16.1	12	14.3	
Drug abuse	Yes	*n*.*a*					6	3.0	12	14.3	0.002 (0.002)
	No						152	76.4	60	71.4	
	Missing						41	20.6	12	14.3	
Weapons possession	Yes	*n*.*a*					137	68.8	64	76.2	0.271 (0.271)
	No						62	31.2	20	23.8	
	Missing						0	0.0	0	0.0	

p* values presented for both imputed (former) and raw model (latter); *n*.*a*.–not available

**Table 2:** Relationship between the couple, and children exposure to IPV.

		G1 (n = 199)		G2 (n = 84)		p*
		*n*	*%*	*n*	*%*	
Relationship between the couple	Current	187	94.0	73	86.9	0.045 (0.045)
	Past	12	6.0	11	13.1	
Couple current relationship	Married	153	76.9	57	67.9	0.113 (0.113)
	Unmarried	46	23.1	27	32.1	
Children’s exposure to IPV	Yes	107	53.8	34	40.5	0.045 (0.045)
	No	64	32.2	41	48.8	
	Missing	28	14.1	9	10.7	

p* values presented for both imputed (former) and raw model (latter)

The types of violence registered are described in Table 3. Physical and psychological abuse were the most frequent forms of violence in both groups, the latter being considered as such only when isolated. No significant statistical differences were found between groups.

**Table 3:** Type of inflicted abuse by the defendant.

		G1 (n = 199)		G2 (n = 84)		p*
		*n*	*%*	*n*	*%*	
Physical	Yes	147	73.9	53	63.1	0.258 (0.258)
	No	41	20.6	22	26.2	
	Missing	11	5.5	9	10.7	
Psychological/emotional	Yes	141	70.9	61	72.6	0.349 (0.349)
	No	47	23.6	14	16.7	
	Missing	11	5.5	9	10.7	
Sexual	Yes	6	3.0	1	2.4	*n*.*a*
	No	180	90.5	73	86.9	
	Missing	13	6.5	9	10.7	
Economic	Yes	22	11.1	7	8.3	0.620 (0.646)
	No	157	78.9	68	81.0	
	Missing	20	10.1	9	10.7	
Social isolation	Yes	20	10.1	9	10.7	0.999 (0.991)
	No	161	80.9	66	78.6	
	Missing	18	9.0	9	10.7	

* p values presented for both imputed (former) and raw model (latter); *n*.*a*.–not applicable

The factors identified as determinants for DGRSP referral are described in Table 4. The defendant’s unemployment and drug abuse were the only variables with a significant statistical association with the state prosecutor’s referral of the defendant to DGRSP.

**Table 4:** Determinants for DGRSP/SRP referral.

			OR	[95% CI]	*p*
Age	Victim		1.06	[1.01–1.12]	0.603
	Defendant		0.98	[0.89–1.07]	0.588
Relationship between the couple	Married	Current	3.39	[0.47–24.57]	0.222
		Past	2.69	[0.09–77.10]	0.588
Unemployment	Victim		0.83	[0.31–2.22]	0.715
	**Defendant**		**3.18**	**[1.19–8.5]**	**0.022**
Type of abuse inflicted	Physical		2.23	[0.76–6.57]	0.144
	Psychological/emotional		0.93	[0.28–3.11]	0.903
	Sexual		0.37	[0.02–6.17]	0.188
	Economic		4.31	[0.06–31.02]	0.146
	Social isolation		0.29	[0.05–1.84]	0.188
Drug and alcohol consumption and other risk factors	Alcohol abuse		0.65	[0.25–1.68]	0.370
	**Drug abuse**		**0.06**	**[0.01–0.51]**	**0.010**
	Weapon possession		1.47	[0.05–43.10]	0.861
	Children’s exposure to IPV		2.37	[0.79–7.16]	0.124

OR—Odds Ratio; CI—confidence interval

## Discussion

The sociodemographic characteristics of victims and defendants in the study sample are similar to those of the original sample (*n* = 1662—Fig 2) [8, 26], before narrowing to PSCP cases only. These characteristics are also similar to those of other studies [35, 37, 42–44].

### Referral by CJS for SRP

Regarding IPV offenders, conviction rarely occurs [9–12]. In Portugal, in 2020, only 14.9% of the defendants were prosecuted and, of these, 2.4% were convicted [45]–and 17.2% serve a prison sentence [46]. This data is considerably lower when compared to other countries. For example, data from the United States and Canada has shown that 60% of IPV court cases result in conviction [47].

However, imprisonment has revealed to have little efficacy in revictimization and recidivism deterrence [8, 13, 14], not to mention other costs associated with the imprisonment process [13, 15].

Considering SRP, findings of several recent meta-analytic studies on its effectiveness on recurrence and recidivism with IPV offenders, point to a small-scale effect [48–51]. However, SRP has shown encouraging results [36] compared to incarceration, although more evidence on their efficacy is required [48, 51]. This shortcoming may be due to the argument that one-size-fits-all treatment programs, as those used on IPV offenders, disregard differences in the interaction factors [52, 53]. Yet, it is noteworthy that, although small, this impact has proven significant in the lives of many victims [54, 55]. Overall, mandatory SRP with an intervention focused on social factors and challenging the defendant’s dysfunctional beliefs can be effective in reducing the likelihood of recidivism and protecting victims.

In the present study, considering the initial sample (*n* = 1662), only 11.9% of the individuals were assigned to attend SRP under DGRSP (*n* = 199). Yet, of those who were subject to PSCP implementation (*n* = 283; 17%), 70.3% were forwarded to SRP. Considering these figures, it poses as relevant to investigate which possible criteria are determining the state prosecutors’ decisions in these cases.

### Determinants for SRP referral

Understanding which variables play a key role in the decision-making process of state prosecutors when referring a defendant to SRP is critical. Ideally, a holistic assessment of both offender and victim characteristics should be carried out [35–38]. Yet, the literature points more commonly to factors related more specific to defendants, including [35–37, 56–58]: age; population affiliation; education level; employment status; personality characteristics; substance abuse history; criminal history; the number of prior IPV cases; type of inflicted violence; interpersonal relationship with the victim; children exposure to IPV. It imposes a reflection on each of these possible determinants:

- **Age**: Regarding the defendant’s age, no differences were found in the current study. However, other authors have suggested that older defendants are more likely to complete an SRP [59], and perhaps more open to welcoming alternative strategies to IPV deterrence [26], which could weigh on the CJS decision. On other hand, younger age is considered an individual and environmental risk factor for recidivism [60]. Considering the victim’s age, differences were found between groups (*χ*^2^ = 18.81, *p* = 0.043), however not exposing it as a determinant of the state prosecutor’s decision for SRP.
- **Population affiliation**: This was not considered in the present study since it was not available. Due to cultural characteristics, it may affect the effectiveness of the SRP efforts to change an offender’s behaviour [61], but it is insufficient for facilitators to adjust to the offenders’ culture [62]. The programs themselves must be adapted to these differences [37, 38, 63].
- **Education level**: This information was also not available in our data, despite its relevance. The offender’s education may be a predicting factor in determining who completes or drops out of treatment programs. Offenders who drop out of SRP demonstrated to have less education than those who completed the program [37, 44, 59, 64]. The offender’s ability to grasp the concepts in the SRP may lead to increased motivation, and more dedicated involvement in the working sessions [37, 44].
- **Employment status**: Our data analysis revealed that the likelihood of being referred to SRP was tripled in cases of unemployment as compared to employed defendants. Such a finding poses it as a determinant in the CJS decision-making (Table 4). This is aligned with research that found that a fourth to a third of individuals currently enrolled in SRP are unemployed [47]. Furthermore, unemployment and low income are considered as individual and environmental risk factors for recidivism and to predict IPV [60, 65, 66], as unemployment frequently generates problems within the couple [67]. Thus, it is expected that the state prosecutor may assume that an unemployed defendant would spend more time at home and therefore be more likely to commit new aggressions. Considering the victim, our results didn’t present any significant difference between groups. This may be because of the state prosecutor’s strong focus on the defendant profile. However, it is known that in cases of female victims, unemployment creates an economic dependency on the offender, a condition that may put the victim in a vulnerable situation being considered an IPV risk liability [66, 68, 69].
- **Personality characteristics**: These aspects were not studied, because in Portugal they are not typically collected by the CJS, mostly due to a data protection law that prohibits the presentation of ethnic data. However, literature reveals that the defendant’s personality characteristics may interact differently with the SRP [63] and can affect the effectiveness of the program’s efforts to change the offender´s behaviour [38]. Thus, this should always be considered to support the state prosecutor’s decision-making. Regardless, in Portugal, the offender’s psychosocial characteristics are always considered in the intervention by the DGRSP.
- **Substance abuse**: Regarding drug abuse, our results show differences between groups (*χ*^2^ = 17.47, *p* = 0.002), and revealed that defendants with a history of drug abuse are less likely than those without such a history to attend SRP (Table 4). Therefore, not being a drug user may be considered as a determinant for state prosecutor decisions. This may be explained by the necessity of sending the addicted defender to other programs for more suitable care, namely for substance abuse addiction therapy [70–72]. However, regarding alcohol abuse, no differences between groups were found, perhaps because alcohol consumption is socially accepted in Portugal [73] and in certain cases, the difference between alcohol consumption and alcohol abuse is not so clear. Literature states that alcohol, like drug abuse, is considered an individual and environmental risk factor for recidivism and increases the number of occurrences and the severity of IPV events [43, 56–58, 65]. Excessive alcohol consumption increases the risk of physical/psychological abuse by eight times and doubles the risk of intimate partner homicide (attempted or consummated murder). Thus, it seems that this factor should be taken into consideration by the state prosecutor.
- **Criminal history**: Criminal history was not included in the study from our initial research [26], because of the scarce number of cases that allowed us to obtain reliable results. Evidence from several studies found that IPV offenders had high rates of prior criminal history [74, 75], particularly in intimate partner homicide (IPH) cases [76]. The cases for weapon possession by the defendant were analyzed, verifying the inexistence of differences between groups and that it was not a determinant in mandatory SRP. However, weapon possession, namely firearms, among male partners with a history of IPV is a lethal risk factor for the IPH [77]. It doubles the risk of an intimate partner´s attempted or consummated murder [56–58, 65, 76, 78]. Such an outcome may be dependent on the state prosecutor´s assessment of the risk levels in the case and consideration of applying other heavier, and hazard-adjusted legal measures.
- **The number of prior IPV cases**: This information was also unavailable for the study, despite its significance. While the tendency for violence to decrease after the alleged offenders’ first entry into the CJS, violence seems to be more severe and frequent in re-entries cases [8, 79]. Previous IPV cases are one of the most common risk factors of IPH [58, 76, 78, 80]. Considering “*The best predictor of crime is prior criminal behaviour”* [81], this factor should be always be considered in the CJS decision.
- **Type of inflicted violence**: The present study found no differences between groups, in this case, a factor which also does not appear to be a determinant for SPR attendance. It is conceivable that these results are due to the state prosecutor´s experience in previous cases devaluing the type of violence suffered by the victim [34]. However, attention should be given to the fact that certain types of IPV which are associated with IPH, may include harassment, physical violence, namely with strangulation, sexual violence, and child exposure to IPV [58, 77, 82–84].
- **Couple relationship:** Differences were found between groups (*χ*^2^ = 6.19, *p* = 0.045) but this aspect did not show to be a determinant in the CSJ decision. However, literature states that separated couples, or those living apart, have a greater probability of being referred to SRP since this aspect is considered one of the strongest predictors of recidivism [24, 71, 85].
- **Children exposure to IPV:** Differences were found between groups (*χ*^2^ = 9.75, *p* = 0.045) but this aspect did not prove to be a determinant factor in the state prosecutor’s decision-making process. Nevertheless, the present study reveals a concerning number of children exposed to IPV (more than 53%). Exposure of children to violence between parents or caregivers is still a much-researched risk factor [86–88]. This aspect must be taken into account, as it constitutes a form of serious violence against children, a risk factor for IPH, and prevention and rehabilitation of IPV cases need to include all involved family members [88–91].

### Study limitations and avenues for further reviews

The present results must be read with caution considering: (a) the low dimension of the sample; (b) data were obtained through IPV police reports and filled in by information provided by the parties involved; (c) data refers only to IPV crimes committed in the district of Porto, in the north of Portugal, and different outcomes may arise in other regions of this country or other countries; (d) some risk factors with dynamic characteristics (e.g. drug and alcohol consumption; couple relationship status; employment status) may have changed during the time of the data collection (police report) and the decision on whether to refer the case to SRP; (e) some important factors, mentioned above, were not studied due to lack of information or lack of a sufficient number of cases for this purpose.

Further studies should consider: (a) larger samples; (b) other samples, such as women perpetrators, different ethnic groups, and the LGBTIQ+ population; (c) a qualitative approach by interviewing state prosecutors, which may provide a wider content analysis of their decision-making process and allow for a better understanding of what underlies such decisions regarding which cases should be referred to SRP.

## Conclusions

This study allows the following main conclusions to be drawn:

- There were differences between groups regarding the following: victim’s age; couple living in a current relationship; drug-addicted defendant; IPV child exposure;
- Unemployment and drug abuse showed to be determinant variables for state prosecutor decisions, regarding defendant referral to SRP attendance.

This study allows us to further consider that:

- The effectiveness of state prosecution services would benefit from systematically taking into account all IPV risk factors substantiated by scientific evidence, instead of leaving it as a burden on the decision-maker;
- This effectiveness would also increase if the reintegration solutions used were based on the needs of each individual, rather than a “one-size-fits-all” approach;
- The creation of an index or checklist based on what science reveals useful in IPV offenders’ reintegration might support the state prosecutor’s decision-making processes, as long as individuals involved are always looked at from an ecological approach. This Ecological Model [92, 93] has been adapted to explain social phenomena, in IPV cases, the risk for violence victimisation and violence perpetration. In the Ecological Model, personal, situational, and sociocultural factors are interpreted to understand how IPV may result from the interaction of factors at different levels of the social environment [94].

## References

[1] Garcia-Moreno C, Jansen HA, Ellsberg M, Heise L, Watts CH. Prevalence of intimate partner violence: findings from the WHO multi-country study on women’s health and domestic violence. The Lancet. 2006;368(9543):1260–9. http://10.1016/S0140-6736(06)69523-8. 10.1016/S0140-6736(06)69523-8 17027732

[2] FRA EUAfFR-. Violence against women: An EU-wide survey: Main results. Austria: FRA, European Union Agency for Fundamental Rights, 2014 9292393421.

[3] Goodman L, Epstein D. Listening to battered women: A survivor-centered approach to advocacy, mental health, and justice. Washington: American Psychological Association; 2008.

[4] Voith LA, Logan-Greene P, Strodthoff T, Bender AE. A Paradigm Shift in Batterer Intervention Programming: A Need to Address Unresolved Trauma. Trauma, Violence, & Abuse. 2020;21(4):691–705. doi: 10.1177/1524838018791268 . 30060720

[5] Feder L, Wilson DB. A meta-analytic review of court-mandated batterer intervention programs: Can courts affect abusers’ behavior? Journal of Experimental Criminology. 2005;1(2):239–62. doi: 10.1007/s11292-005-1179-0

[6] Scott K, King C, McGinn H, Hosseini N. Effects of Motivational Enhancement on Immediate Outcomes of Batterer Intervention. Journal of Family Violence. 2011;26(2):139–49. doi: 10.1007/s10896-010-9353-1

[7] Buttell FP, Powers D, Wong A. Evaluating Predictors of Program Attrition Among Women Mandated Into Batterer Intervention Treatment. Research on Social Work Practice. 2012;22(1):20–8. doi: 10.1177/1049731511413473

[8] Vieira-Pinto P, Muñoz-Barús José I, Taveira-Gomes T, Vidal-Alves MJ, Magalhães T. Intimate partner violence against women. Does violence decrease after the entry of the alleged offender into the criminal justice system? Forensic Sciences Research. 2021:1–8. doi: 10.1080/20961790.2021.1960616 35341122 PMC8942538

[9] Sherman LW. Domestic violence and restorative justice: Answering key questions. Va J Soc Pol’y & L. 2000;8:263.

[10] Garner JH, Maxwell CD. Prosecution and conviction rates for intimate partner violence. Criminal Justice Review. 2009;34(1):44–79.

[11] Dichter ME, Cerulli C, Kothari CL, Barg FK, Rhodes KV. Engaging With Criminal Prosecution: The Victim’s Perspective. Women & Criminal Justice. 2011;21(1):21–37. doi: 10.1080/08974454.2011.536053

[12] Bell Cattaneo, Goodman Dutton. Criminal Case Outcomes, Incarceration, and Subsequent Intimate Partner Violence. Journal of Family Violence. 2013;28(5):489–502. doi: 10.1007/s10896-013-9515-z WOS:000320314300007.

[13] Cullen FT, Jonson CL, Nagin DS. Prisons Do Not Reduce Recidivism:The High Cost of Ignoring Science. The Prison Journal. 2011;91(3_suppl):48S–65S. doi: 10.1177/0032885511415224

[14] Castro Rodrigues A, Sacau A, Oliveira JQ, Gonçalves RA. Prison sentences: last resort or the default sanction? Psychology, Crime & Law. 2019;25(2):171–94. doi: 10.1080/1068316X.2018.1511788

[15] Bushnell A. Skewed Priorities: Comparing the growth of prison spending with police spending. 2019.

[16] Sutton A, Cherney A, White R, Clancey G. Crime prevention: Principles, perspectives and practices: Cambridge University Press; 2021.

[17] Capdevila M, Ferrer M, Framis B, BLANCH M, Garrigós A, Batlle A, et al. La reincidencia en medidas penales alternativas, 2015. Barcelona: Centre d’Estudis Jurídics i Formació Especialitzada. 2016.

[18] Dandurand Y. Alternative Approaches to Preventing Recidivism: Restorative Justice and the Social Reintegration of Offenders. Women and Children as Victims and Offenders: Background, Prevention, Reintegration: Springer; 2016. p. 283–99.

[19] Landenberger NA, Lipsey MW. The positive effects of cognitive–behavioral programs for offenders: A meta-analysis of factors associated with effective treatment. Journal of experimental criminology. 2005;1(4):451–76.

[20] Wilson DB, Bouffard LA, MacKenzie DL. A quantitative review of structured, group-oriented, cognitive-behavioral programs for offenders. Criminal Justice and Behavior. 2005;32(2):172–204.

[21] Easton CJ, Crane CA, Mandel D. A randomized controlled trial assessing the efficacy of cognitive behavioral therapy for substance‐dependent domestic violence offenders: an integrated substance abuse‐domestic violence treatment approach (SADV). Journal of marital and family therapy. 2018;44(3):483–98. doi: 10.1111/jmft.12260 29108096

[22] UNODC. United Nations Office on Drugs and Crime—Prevention of Recidivism and the Social Reintegration of Offenders. United Nations. 2018;CRIMINAL JUSTICE HANDBOOK SERIES UNITED NATIONS Vienna,:138.

[23] Connors AD, Mills JF, Gray AL. Intimate partner violence intervention for high-risk offenders. Psychological Services. 2013;10(1):12. doi: 10.1037/a0028979 22924803

[24] Bonta J, Andrews DA. Viewing offender assessment and rehabilitation through the lens of the risk-need-responsivity model. Offender Supervision: New Directions in Theory, Research and Practice. 2010:19–40.

[25] Andrews DA, Zinger I, Hoge RD, Bonta J, Gendreau P, Cullen FT. Does correctional treatment work? A clinically relevant and psychologically informed meta‐analysis. Criminology. 1990;28(3):369–404.

[26] Vieira-Pinto P, Muñoz-Barús JI, Taveira-Gomes T, Vidal-Alves MJ, Magalhães T. Suspension of Criminal Proceedings for Perpetrators of Intimate Partner Violence Against Women: Impact on Re-Entries. Front Psychol. 2021;12(4914). doi: 10.3389/fpsyg.2021.725081 34777104 PMC8586086

[27] DGRSP. Relatório Estatístico Anual—Assessoria Técnica à Tomada de Decisão Judicial (Relatórios e Audições) e Execução de Penas e Medidas nas áreas Penal e Tutelar Educativa. Direção Geral de Reinserção e Serviços Prisionais—Ministério da Justiça, 2018 julho 2019. Report No.

[28] Rijo D, Capinha M. A reabilitação dos agressores conjugais: dos modelos tradicionais de reabilitação ao Programa Português para Agressores de Violência Doméstica (PAVD). Ousar Integrar. 2012;11:83–97.

[29] GREVIO. GREVIO reports, relevant for programmes for perpetrators of violence https://www.coe.int/en/web/istanbul-convention/country-monitoring-work: Council of Europe; 2021 [cited 2021 2021-09-16]. Available from: https://www.coe.int/en/web/istanbul-convention/country-monitoring-work.

[30] Quintas J, Fonseca E, Sousa H, Serra A. Programa para agressores de violência doméstica: Avaliação do impacto da aplicação experimental (2010–2011)[Program for Domestic Violence Offenders: Assessing the Impact of Experimental Application (2010–2011)]. Ousar integrar–revista de reinserção social e prova. 2012;12:9–26.

[31] Manita C. A intervenção em agressores no contexto da violência doméstica em Portugal: Estudo preliminar de caracterização.[Preliminary study of the characterization of the intervention with offenders in the context of marital violence in Portugal]. Lisboa: Comissão para a Igualdade e para os Direitos das Mulheres; 2005. 115 p.

[32] Cunha O, Gonçalves RA. Tratamento de agressores domésticos: O Programa de promoção e intervenção com agressores conjugais (ppriac). Anuario de Psicología Jurídica. 2011;16:41–64.

[33] Hamilton L, Koehler JA, Lösel FA. Domestic Violence Perpetrator Programs in Europe, Part I:A survey of Current Practice. International Journal of Offender Therapy and Comparative Criminology. 2013;57(10):1189–205. doi: 10.1177/0306624X12469506 . 23267241

[34] Jamal S, Manita C. O recurso à suspensão provisória do processo em crimes de violência doméstica: Perceções e decisões dos/as magistrados/as. Suspended prosecution in domestic violence crimes: prosecutors’ perceptions and decisions. 2017;31(2):297–300. .

[35] Bennett LW, Stoops C, Call C, Flett H. Program Completion and Re-Arrest in a Batterer Intervention System. Research on Social Work Practice. 2007;17(1):42–54. doi: 10.1177/1049731506293729

[36] Gondolf EW, Bennett L, Mankowski E. Lessons in Program Evaluation: The ACTV Batterer Program Study and Its Claims. Violence Against Women. 2019;25(5):NP1–NP10. doi: 10.1177/1077801217741994 . 29361885

[37] Jewell LM, Wormith JS. Variables associated with attrition from domestic violence treatment programs targeting male batterers: A meta-analysis. Criminal Justice and Behavior. 2010;37(10):1086–113. doi: 10.1177/0093854810376815

[38] Spiropoulos GV, Salisbury EJ, Van Voorhis P. Moderators of Correctional Treatment Success:An Exploratory Study of Racial Differences. International Journal of Offender Therapy and Comparative Criminology. 2014;58(7):835–60. doi: 10.1177/0306624X13492999 . 23824087

[39] Team RC. R: A language and environment for statistical computing. R Foundation for Statistical Computing. 2014.

[40] Little RJ. A test of missing completely at random for multivariate data with missing values. Journal of the American statistical Association. 1988;83(404):1198–202.

[41] Van Buuren S. Flexible imputation of missing data: Chapman and Hall/CRC; 2018.

[42] Cunha OS, Gonçalves RA. Efficacy Assessment of an Intervention Program With Batterers. Small Group Research. 2015;46(4):455–82. doi: 10.1177/1046496415592478

[43] Cunha OS, Goncalves RA. Predictors of Intimate Partner Homicide in a Sample of Portuguese Male Domestic Offenders. J Interpers Violence. 2019;34(12):2573–98. Epub 2016/08/10. doi: 10.1177/0886260516662304 . 27503324

[44] Buttell FP, Carney MM. A Large Sample Investigation of Batterer Intervention Program Attrition: Evaluating the Impact of State Program Standards. Research on Social Work Practice. 2008;18(3):177–88. doi: 10.1177/1049731508314277

[45] RASI. Relatório Anual de Segurança Interna—2020. Lisboa: Ministério da Administração Interna; 2020.

[46] CIG. Indicadores Estatisticos Lisboa2021 [updated July, 2021; cited 2021 17-11-2021]. Available from: https://www.cig.gov.pt/area-portal-da-violencia/portal-violencia-domestica/indicadores-estatisticos/.

[47] Cannon C, Hamel J, Buttell F, Ferreira RJ. A Survey of Domestic Violence Perpetrator Programs in the United States and Canada: Findings and Implications for Policy and Intervention. Partner Abuse. 2016;(3):226–76. doi: 10.1891/1946-6560.7.3.226

[48] Arce R, Arias E, Novo M, Fariña F. Are Interventions with Batterers Effective? A Meta-analytical Review. Psychosocial Intervention. 2020. 10.5093/pi2020a11.

[49] Travers Á, McDonagh T, Cunningham T, Armour C, Hansen M. The effectiveness of interventions to prevent recidivism in perpetrators of intimate partner violence: A systematic review and meta-analysis. Clinical Psychology Review. 2021;84:101974. doi: 10.1016/j.cpr.2021.101974 33497921

[50] Karakurt G, Koç E, Çetinsaya EE, Ayluçtarhan Z, Bolen S. Meta-analysis and systematic review for the treatment of perpetrators of intimate partner violence. Neuroscience & Biobehavioral Reviews. 2019;105:220–30. Epub 2019/08/16. doi: 10.1016/j.neubiorev.2019.08.006 ; PubMed Central PMCID: PMC6742529. 31415863 PMC6742529

[51] Arias E, Arce R, Vilariño M. Batterer intervention programmes: A meta-analytic review of effectiveness. Psychosocial Intervention. 2013;22(2):153–60. doi: 10.5093/in2013a18

[52] Cunha O, Gonçalves RA. Intimate partner violence offenders: Generating a data-based typology of batterers and implications for treatment. The European Journal of Psychology Applied to Legal Context. 2013;5(2):131–9. 10.5093/ejpalc2013a2.

[53] Novo M, Fariña F, Seijo MD, Arce R. Assessment of a community rehabilitation programme in convicted male intimate-partner violence offenders1. International Journal of Clinical and Health Psychology. 2012;12(2):219–34. .

[54] Babcock JC, Green CE, Robie C. Does batterers’ treatment work? A meta-analytic review of domestic violence treatment. Clin Psychol Rev. 2004;23(8):1023–53. Epub 2004/01/20. doi: 10.1016/j.cpr.2002.07.001 . 14729422

[55] Babcock J, Armenti N, Cannon C, Lauve-Moon K, Buttell F, Ferreira R, et al. Domestic Violence Perpetrator Programs: A Proposal for Evidence-Based Standards in the United States. Partner Abuse. 2016;(4):355–460. doi: 10.1891/1946-6560.7.4.355

[56] Foran HM, O’Leary KD. Alcohol and intimate partner violence: A meta-analytic review. Clinical psychology review. 2008;28(7):1222–34. Epub 2008/06/14. doi: 10.1016/j.cpr.2008.05.001 . 18550239

[57] Moore TM, Stuart GL, Meehan JC, Rhatigan DL, Hellmuth JC, Keen SM. Drug abuse and aggression between intimate partners: A meta-analytic review. Clinical psychology review. 2008;28(2):247–74. Epub 1999/08/27. doi: 10.1016/j.cpr.2007.05.003 . 17604891

[58] Spencer CM, Stith SM. Risk Factors for Male Perpetration and Female Victimization of Intimate Partner Homicide: A Meta-Analysis. Trauma Violence Abuse. 2020;21(3):527–40. Epub 2018/06/12. doi: 10.1177/1524838018781101 . 29888652

[59] Buttell FP, Carney MM. Psychological and demographic predictors of attrition among batterers court ordered into treatment. Social Work Research. 2002;26(1):31–41. doi: 10.1093/swr/26.1.31

[60] Smith ME. Self-Deception Among Men Who Are Mandated to Attend a Batterer Intervention Program. Perspectives in Psychiatric Care. 2007;43(4):193–203. doi: 10.1111/j.1744-6163.2007.00134.x 17894669

[61] White RJ, Gondolf EW. Implications of personality profiles for batterer treatment. Journal of Interpersonal Violence. 2000;15(5):467–88.

[62] Gondolf EW. Culturally-Focused Batterer Counseling for African-American Men*. Criminology & Public Policy. 2007;6(2):341–66. 10.1111/j.1745-9133.2007.00441.x.

[63] Bonta J, Andrews DA. The psychology of criminal conduct. 6th ed ed: Routledge; 2016.

[64] Stalans LJ, Seng M. Identifying subgroups at high risk of dropping out of domestic batterer treatment: The buffering effects of a high school education. International Journal of Offender Therapy and Comparative Criminology. 2007;51(2):151–69. doi: 10.1177/0306624X06290204 17412821

[65] Campbell JC, Webster DW, Glass N. The Danger Assessment: Validation of a Lethality Risk Assessment Instrument for Intimate Partner Femicide. Journal of Interpersonal Violence. 2008;24(4):653–74. Epub 2008/08/01. doi: 10.1177/0886260508317180 . 18667689 PMC7878014

[66] Campbell JC. Health consequences of intimate partner violence. The Lancet. 2002;359(9314):1331–6. doi: 10.1016/S0140-6736(02)08336-8 11965295

[67] Stets JE. Job autonomy and control over one’s spouse: a compensatory process. J Health Soc Behav. 1995;36(3):244–58. 7594357

[68] Messing JT, O’Sullivan CS, Cavanaugh CE, Webster DW, Campbell J. Are abused women’s protective actions associated with reduced threats, stalking, and violence perpetrated by their male intimate partners? Violence against women. 2017;23(3):263–86. doi: 10.1177/1077801216640381 27118689

[69] Bornstein RF. The complex relationship between dependency and domestic violence: Converging psychological factors and social forces. Am Psychol. 2006;61(6):595. doi: 10.1037/0003-066X.61.6.595 16953747

[70] Vitoria-Estruch S, Romero-Martínez A, Lila M, Moya-Albiol L. Differential cognitive profiles of intimate partner violence perpetrators based on alcohol consumption. Alcohol. 2018;70:61–71. doi: 10.1016/j.alcohol.2018.01.006 29800781

[71] Lila M, Martin-Fernandez M, Gracia E, Lopez-Ossorio JJ, Gonzalez JL. Identifying Key Predictors of Recidivism among Offenders Attending a Batterer Intervention Program: A Survival Analysis. Psychosocial Intervention. 2019;28(3):157–67. doi: 10.5093/pi2019a19 WOS:000499111500006.

[72] Kraanen FL, Vedel E, Scholing A, Emmelkamp PM. The comparative effectiveness of integrated treatment for substance abuse and partner violence (I-StoP) and substance abuse treatment alone: a randomized controlled trial. BMC psychiatry. 2013;13(1):1–14. 24059784 10.1186/1471-244X-13-189 PMC3716952

[73] Silva Ana Patricia, et al. "Cheers, proost, saúde: Cultural, contextual and psychological factors of wine and beer consumption in Portugal and in the Netherlands." Critical reviews in food science and nutrition 57.7 (2017): 1340–1349. doi: 10.1080/10408398.2014.969396 26560863

[74] Maxwell CD, Garner JH, Fagan JA. The Preventive Effects of Arrest on Intimate Partner Violence: Research, Policy and Theory*. Criminology & Public Policy. 2002;2(1):51–80. 10.1111/j.1745-9133.2002.tb00107.x.

[75] Ventura LA, Davis G. Domestic violence court case conviction and recidivism. Violence Against Women. 2005;11(2):255–77. doi: 10.1177/1077801204271722 16043549

[76] Zeoli AM, Kwiatkowski CC, Wallin MA, Brown K. Criminal Histories of Intimate Partner Homicide Offenders. Homicide Studies. 2021;0(0):10887679211046866. doi: 10.1177/10887679211046866

[77] Campbell JC, Webster D, Koziol-McLain J, Block C, Campbell D, Curry MA, et al. Risk factors for femicide in abusive relationships: results from a multisite case control study. Am J Public Health. 2003;93(7):1089–97. doi: 10.2105/ajph.93.7.1089 . 12835191 PMC1447915

[78] Cunha OS, Gonçalves RA. Predictors of Intimate Partner Homicide in a Sample of Portuguese Male Domestic Offenders. Journal of Interpersonal Violence. 2019;34(12):2573–98. doi: 10.1177/0886260516662304 . 27503324

[79] Bland M, Ariel B. Targeting escalation in reported domestic abuse: Evidence from 36,000 callouts. International criminal justice review. 2015;25(1):30–53. doi: 10.1177/1057567715574382 WOS:000438937800003.

[80] Pereira AR, Vieira DN, Magalhães T. Fatal intimate partner violence against women in Portugal: A forensic medical national study. Journal of forensic and legal medicine. 2013;20(8):1099–107. doi: 10.1016/j.jflm.2013.09.015 24237830

[81] Hirschi T. Self-control and crime. In: Press TG, editor. Handbook of self-regulation Research, Theory, and Applications. 3rd Edition ed. New York: The Guilford Press; 2017. p. 538–52.

[82] Messing JT, Campbell J, Wilson JS, Brown S, Patchell B. The Lethality Screen: The Predictive Validity of an Intimate Partner Violence Risk Assessment for Use by First Responders. Journal of Interpersonal Violence. 2017;32(2):205–26. doi: 10.1177/0886260515585540 WOS:000393675900004. 25969441

[83] Campbell JC, Glass N, Sharps PW, Laughon K, Bloom T. Intimate Partner Homicide: Review and Implications of Research and Policy. Trauma, Violence, & Abuse. 2007;8(3):246–69. doi: 10.1177/1524838007303505 17596343

[84] Stith SM, Smith DB, Penn CE, Ward DB, Tritt D. Intimate partner physical abuse perpetration and victimization risk factors: A meta-analytic review. Aggression and Violent Behavior. 2004;10(1):65–98. 10.1016/j.avb.2003.09.001.

[85] Crowley L. Domestic Violence Perpetrator Programmes in Ireland–Intervention Required! International Journal of Law, Policy and the Family. 2017;31(3):291–310. doi: 10.1093/lawfam/ebx010 WOS:000419221400002.

[86] Carter B, Paranjothy S, Davies A, Kemp A. Mediators and Effect Modifiers of the Causal Pathway Between Child Exposure to Domestic Violence and Internalizing Behaviors Among Children and Adolescents: A Systematic Literature Review. Trauma Violence Abuse. 2020;0(0):1524838020965964. Epub 2020/10/24. doi: 10.1177/1524838020965964 . 33094689 PMC8905123

[87] Li S, Zhao F, Yu G. Childhood maltreatment and intimate partner violence victimization: A meta-analysis. Child abuse & neglect. 2019;88:212–24. doi: 10.1016/j.chiabu.2018.11.012 30537622

[88] Kitzmann KM, Gaylord NK, Holt AR, Kenny ED. Child witnesses to domestic violence: a meta-analytic review. J Consult Clin Psych. 2003;71(2):339. doi: 10.1037/0022-006x.71.2.339 12699028

[89] Bullock L, Ghazarian S, Nimer M, Signing L, Herbell K, Farje D, et al. Children Exposed to IPV: Impact of Multiple Father Figures. Maternal and Child Health Journal. 2021. doi: 10.1007/s10995-021-03184-6 34151395

[90] Doelman EH, Luijk MP, Haen Marshall I, Jongerling J, Enzmann D, Steketee MJ. The association between child maltreatment and juvenile delinquency in the context of Situational Action Theory: Crime propensity and criminogenic exposure as mediators in a sample of European youth? European Journal of Criminology. 0(0):14773708211013300. doi: 10.1177/14773708211013300

[91] Besemer S, Ahmad SI, Hinshaw SP, Farrington DP. A systematic review and meta-analysis of the intergenerational transmission of criminal behavior. Aggression and Violent Behavior. 2017;37:161–78. 10.1016/j.avb.2017.10.004.

[92] Heise Lori L. "Violence against women: An integrated, ecological framework." Violence against women 4.3 (1998): 262–290. doi: 10.1177/1077801298004003002 12296014

[93] Meinhart Melissa, et al. "Identifying the impact of intimate partner violence in humanitarian settings: using an ecological framework to review 15 years of evidence." International journal of environmental research and public health 18.13 (2021): 6963. doi: 10.3390/ijerph18136963 34209746 PMC8297014

[94] Fulu Emma, and Miedema Stephanie. "Violence against women: globalizing the integrated ecological model." Violence against women 21.12 (2015): 1431–1455. 4. doi: 10.1177/1077801215596244 26215287 PMC4638316

